# The Long Pentraxin PTX3 in Bone Homeostasis and Pathology

**DOI:** 10.3389/fimmu.2019.02628

**Published:** 2019-11-08

**Authors:** Raffaella Parente, Cristina Sobacchi, Barbara Bottazzi, Alberto Mantovani, Danka Grčevic, Antonio Inforzato

**Affiliations:** ^1^Department of Immunology and Inflammation, Humanitas Clinical and Research Institute - IRCCS, Milan, Italy; ^2^CNR-IRGB, Milan Unit, Milan, Italy; ^3^Department of Biomedical Sciences, Humanitas University, Milan, Italy; ^4^The William Harvey Research Institute, Queen Mary University of London, London, United Kingdom; ^5^Department of Physiology and Immunology, School of Medicine, University of Zagreb, Zagreb, Croatia; ^6^Croatian Institute for Brain Research, School of Medicine, University of Zagreb, Zagreb, Croatia

**Keywords:** pentraxins, PTX3, osteoblasts, osteoimmunology, periodontitis

## Abstract

The innate immune system is equipped with a number of germ-line encoded soluble pattern recognition molecules (PRMs) that collectively mediate the humoral host response to infection and damage in cooperation with cells and tissues of the immune and non-immune compartments. Despite the impressive diversity in structure, source, and regulation across PRMs, these all share remarkably similar functions inasmuch as they recognize microbes and damaged tissues, activate complement, exert opsono-phagocytic activities, and regulate inflammation. The long pentraxin 3 (PTX3) is a prototypic soluble PRM. Long known as a major player in innate immunity, inflammation and matrix remodeling, only recently has PTX3 emerged as a mediator of bone homeostasis in rodents and humans. *Ptx3*-targeted mice exhibit reduced trabecular volume during bone development, and impaired callus mineralization following experimental fracture. The murine gene is expressed *in vivo* by non-hematopoietic periosteal cells in the early phases of fracture healing, and *in vitro* by maturing osteoblasts. Human osteoblasts do express the PTX3 protein, whose levels positively correlate with bone density *in vivo* and osteoblast proliferation and maturation *in vitro*, thus pointing to a role in bone deposition. Contrasting evidence, however, suggest osteoclastogenesis-promoting effects of PTX3, where its expression has been associated with periodontitis, arthritis, and bone metastasis, conditions hallmarked by inflammation and bone resorption. Here, we review past and recent literature on the functions exerted by this long pentraxin in bone biology, with major emphasis on physiological skeletal remodeling, fracture healing, and chronic diseases of the bone.

## Introduction

The innate immune system holds an ancient place in evolution as a frontline mechanism of defense against exogenous and endogenous threats. The innate immune response initiates with recognition of pathogen- and damage-associated molecular patterns (PAMPs and DAMPs, respectively), by cell-borne and soluble mediators (i.e., PRMs), and progresses to pattern-tailored and microenvironment-dependent effector processes whose action extends far beyond pathogen disposal to embrace tissue homeostasis and cancer (1). Pentraxins are a superfamily of soluble PRMs with a multiplicity of functions in infection immunity, inflammation, and tissue remodeling. C-reactive protein (CRP) and serum amyloid P component (SAP) are the “classical” or short pentraxins that share a typical quaternary structure with 5 or 10 identical subunits folding into pentameric rings. CRP and SAP are acute phase proteins in humans and mice, respectively, whose synthesis is primarily raised in the liver in response to the pro-inflammatory cytokine interleukin (IL)-6 (2, 3). Originally identified in the early 1990s as an IL-1β- and tumor necrosis factor (TNF)-α-inducible gene, PTX3 soon became the prototype of long pentraxins, a subfamily of proteins that contain a structurally unrelated N-terminal region in addition to the family-distinctive carboxy-terminal pentraxin domain (4). PTX3 is made by a number of both immune and non-immune cell types upon stimulation with primary pro-inflammatory mediators and microbial components, and exerts non-redundant roles in infection immunity (5), inflammation, and complement-mediated cancerogenesis (6).

An increasing body of evidence points to PTX3 as a key player in extracellular matrix (ECM) remodeling. In this regard, it is long known that *Ptx3*^−/−^ female mice are sub-fertile, due to defective formation of the viscoelastic hyaluronic acid (HA)-rich matrix that surrounds the oocyte in the preovulatory follicle (i.e., the cumulus oophorous complex), where correct assembly of the cumulus matrix is required for fertilization *in vivo* (7, 8). PTX3 acts therein as a nodal molecule to crosslink HA in cooperation with tumor necrosis factor-inducible gene 6 (TNFAIP6, also known as TSG-6) and the heavy chains (HCs) of inter-alpha-trypsin inhibitor (IαI) (9–11). Also, in different mouse models of sterile tissue damage (skin wound, chemical injury of the liver and lung, arterial thrombosis), genetic ablation of *Ptx3* causes aberrant thrombotic responses, increased formation and prolonged duration of the fibrin clot, and enhanced collagen deposition (12–14). Within this frame, PTX3 expression and release is elicited in macrophages and mesenchymal cells by Toll-like receptors (TLRs) and IL-1β stimulation, and the locally released protein has fibrin remodeling and wound healing promoting effects (15). Furthermore, PTX3 has been shown to recognize selected fibroblast growth factors (FGFs), including FGF2 and FGF8b, through its N-terminal domain, and sequester them in the ECM, thus inhibiting their angiogenic and pro-tumorigenic effects *in vitro* and *in vivo* [see (16) for a review].

Bone remodeling is a peculiar instance of ECM turnover (17), where dynamic cell-cell and cell-matrix interactions set in place that involve a number of tissue growth factors, cytokines, and ECM components as well as increasingly acknowledged contributions from innate immune cells and soluble PRMs (18–20). The intimate crosstalk between immune and bone cells as well as soluble molecules is more apparent under conditions of extensive bone regeneration, e.g., after bone fracture or injury, where local acute inflammatory responses are required to initiate and propagate appropriate tissue healing and repair programs (21). Furthermore, infectious diseases of the bone, like periodontitis and osteomyelitis, set the scene for an even tighter cooperation between bone and immune components, as exemplified by the involvement of the complement system in the onset and progression of periodontitis (22).

As paradigmatic humoral PRM and key component of the ECM, PTX3 is emerging as a new mediator of bone physiopathology. Here, we present and discuss the current understanding of this long pentraxin in osteoimmunology, with an emphasis on recent evidence suggesting novel functions in physiological skeletal remodeling, bone healing, and chronic bone diseases (see Tables 1 and 2).

**Table 1:** PTX3 in bone homeostasis and experimental disease models.

**References**	**Species**	**Model**	**Cell lineage/Biological sample**	**Pathophysiological context/experimental condition**	**Biological effect**
Grcevic et al. (23)	Mouse	*in vitro*	ob & oc	ob & oc differentiation	High PTX3 expression in early ob, but low in oc, differentiation
		*in vivo*	Trabecular bone, fracture callus	Bone remodeling	Reduced BFR in *Ptx3^−/−^*mice
				Fracture healing	Reduced callus mineralization in *Ptx3^−/−^* mice
Scimeca et al. (24)	Human	*in vivo* & *ex vivo*	Femoral head biopsy, ob	Osteoporosis	Reduced PTX3 expression in ob from osteoporotic patients
		*in vitro*	ob	Treatment of primary ob from young healthy controls with anti-PTX3 antibody	Altered morphology, reduced RANKL and RUNX2 expression, and reduced mineralization
Zimmermann et al. (25)	Pig	*in vitro*	Bone-derived fibroblasts	Exposure to bone-conditioned medium w/o TGF-β receptor antagonist	Increased PTX3 expression, which is reversed by the TGF-β receptor antagonist
Chiellini et al. (26)	Human	*in vitro*	Multipotent adipose-derived stem cells	Osteogenic and adipogenic induction	Enhanced PTX3 expression as compared to undifferentiated cells, more pronounced during adipogenesis
Lee et al. (27)	Human	*in vitro*	BM-derived stromal cells	Osteogenic induction ± TNF-α	Increased PTX3 expression and secretion in early, but not late, steps of differentiation; further enhanced by TNF-α
	Mouse	*in vivo*	BM	LPS-induced bone loss	Higher PTX3 expression in LPS- versus vehicle-treated mice
		*in vitro*	ob & oc	ob & oc differentiation & function in the presence of exogenous PTX3	No effect on oc and ob differentiation; in early ob, increased RUNX2 and RANKL expression
Keles et al. (28)	Rat	*in vivo*	Gingival tissue & serum	Ligature-induced experimental periodontitis	PTX3 levels correlate with early, not late, phases of disease
Tsuge et al. (29)	Rat	*in vivo*	PDL	Orthodontic tooth movement	PTX3 levels increase at early time points
Garcia et al. (30)	Mouse	*in vivo*	Arthritic joint	STIA	PTX3 levels increase in *Mmp8*^−/−^ mice

*ob, osteoblast; oc, osteoclast; BFR, Bone Formation Rate; BM, Bone Marrow; TGF-β, Transforming Growth Factor-β; PDL, periodontal ligament; STIA, Serum Transfer-Induced Arthritis*.

**Table 2:** PTX3 in human chronic bone diseases.

**References**	**Model**	**Biological sample**	**Pathophysiological context/experimental condition**	**Biological effect**
Pradeep et al. (46) and Fujita et al. (47)	*in vivo*	GCF & plasma	Gingivitis and periodontitis	PTX3 levels increase during disease progression
Gumus et al. (48)	*in vivo*	Saliva & serum	Periodontitis	PTX3 levels correlate with periodontal tissue inflammation
Lakshmanan et al. (49)	*in vivo*	Gingival tissue	Periodontitis	PTX3 concentration is higher in aggressive as compared to chronic periodontitis
Leira et al. (50)	*in vivo*	Serum	Periodontitis-Chronic Migraine (PD-CM)	Increased PTX3 levels as compared to CM without PD
Temelli et al. (58)	*in vivo*	Serum	Coronary Artery Disease (CAD)	PTX3 levels positively correlate with periodontal inflamed surface area (PISA) in CAD(-) groups
Leira et al. (59)	*in vivo*	Serum	Lacunar Infarct (LI)	PTX3 levels positively correlate with PISA in patients with poor prognosis
Surlin et al. (60)	*in vivo*	GCF	Orthodontic tooth movement	PTX3 levels increase at early time points
Luchetti et al. (51)	*in vitro*	Synoviocytes	RA & OA	PTX3 levels increase in OA cells upon TNF-α stimulation, while they are constitutively elevated in RA cells
	*ex vivo*	Synovial tissue	RA & OA	High PTX3 immunoreactivity in RA tissue as compared to controls
Yokota et al. (52)	*in vitro*	FLS	RA	PTX3 expression is inhibited by simvastatin treatment
Satomura et al. (53)	*in vitro*	Synoviocytes	RA	PTX3 expression is induced by serum amyloid A
Padeh et al. (54)	*in vivo*	SF	Juvenile idiopathic arthritis	Higher PTX3 levels associate with disease severity and prognosis
Choi et al. (56)	*in vitro*	GCC	Advanced gastric cancer	PTX3 expression is induced by TNF-α via NF-kB; PTX3 enhances tumor cell migration and macrophage recruitment
Choi et al. (57)	*ex vivo*	Metastatic tissues	Metastatic breast cancer	Elevated PTX3 expression correlates with poor survival
	*in vitro*	BM-BCCL	Metastatic breast cancer	High PTX3 levels. PTX3 silencing prevents BM-BCC migration, macrophage chemotaxis, and oc formation

*GCF, gingival crevicular fluid; PDL, Periodontal Ligament; RA, Rheumatoid Arthritis; OA, Osteoarthritis; FLS, Fibroblast-Like Synoviocytes; SF, Synovial Fluid; GCC, Gastric Cancer Cells; BM-BCCL, Bone Metastatic-Breast Cancer Cell line; Oc, osteoclast*.

## Gene Regulation and Protein Structure

Sequence and regulation of the *PTX3* gene are highly conserved in evolution, which has allowed assessing the pathophysiological roles of this long pentraxin in gene-targeted animals. The human and murine *PTX3* map on chromosome 3, and share a common structural organization with three exons coding for a leader peptide, the N- and C-terminal domains, respectively [see (4–6) and below].

Expression of the gene is promptly induced in a variety of immune and non-immune cell types by inflammatory cytokines (e.g., IL-1β, TNF-α), TLR agonists, microbial moieties (e.g., lipopolysaccharide, LPS, outer membrane protein A, OmpA, lipoarabinomannans), and intact microorganisms [see4 for a review on gene expression]. PTX3 production is also raised in granulosa cells by ovulation promoting hormones, whereby it participates in structuring the cumulus oophorous ECM, as discussed above (8, 9, 11). As opposed to this, transcription of the *PTX3* gene is inhibited by IFN-γ, IL-4, dexamethasone, 1α,25-dihydroxivitamin D3, and prostaglandin E2 (31, 32). Furthermore, PTX3 is constitutively stored as “pre-made” protein in the specific granules of polymorphonuclear cells (PMNs), is released in response to TLR stimulation, and localizes in the neutrophil extracellular traps (NETs) (33). Expression of the human *PTX3* gene is controlled by epigenetic mechanisms, including differential methylation of the promoter region and two enhancers in physiological and inflammatory conditions [see (34) for more details on epigenetic regulation]. We have recently reported that the murine *Ptx3* gene is expressed *in vitro* by maturing osteoblasts and *in vivo* by bone-encased osteocytes (23). Also, PTX3 expression has been documented in human osteoblasts, based on observations from both *in vivo* and *in vitro* studies (24, 26, 27, 35).

The human PTX3 protomer is a 381aa-long glycoprotein that contains a secretion-targeting signal peptide (1–17), an N-terminal region (18–178), and a C-terminal pentraxin domain (179–381). The N-terminal domain sequence is highly divergent from that of proteins with known structure, however, likely contains coiled-coils and intrinsically disordered regions, which are believed to contribute structural and functional versatility to the protein (36). The C-terminal pentraxin domain bears a single N-glycosylation site that is occupied by complex type oligosaccharides with tissue- and stimulus-dependent composition (37) and tuning effects on the protein's function in inflammation and innate immunity (38).

The mature PTX3 protein has a peculiar quaternary structure with eight identical protomer subunits folding into an asymmetric and rather elongated molecule that is stabilized both by disulfide bonds and non-covalent interactions (36). This structural complexity is necessary for the long pentraxin to engage in a number of interactions with a variety of ligands, including microbes, complement and matrix proteins, and thereby accomplish its pleiotropic functions [reviewed in (39)].

## Bone Homeostasis and Fracture Healing

Excessive and uncontrolled inflammation has bone resorbing effects due to suppression of osteoblast and enhancement of osteoclast functions (17–21). On the other hand, proinflammatory mediators are required for physiological bone remodeling, a highly coordinated process that couples bone resorption and formation to maintain structural integrity and metabolic balance, and is regulated by mechanical loading, microdamage, hormonal signals, and local factors (17, 40, 41). Moreover, an inflammatory milieu is necessary to promote tissue regeneration after bone fracture or injury (21, 42, 43). Fracture healing proceeds through sequential steps of inflammation, induced angiogenesis, mesenchymal progenitors recruitment, cartilage and bone formation, extracellular matrix synthesis, and callus remodeling. During this process, a balanced local microenvironment, ensured both by immune and bone cells, is crucial for the beneficial effects of transient acute inflammation on bone regeneration. Amongst the bone-active inflammatory mediators, PTX3 has been shown to participate in bone homeostasis, based on *in vivo, ex vivo* and *in vitro* evidence, which is discussed in the following paragraphs and summarized in Table 1.

### In vivo

Our group has recently shown that genetic ablation of *Ptx3* in the mouse leads to reduced osteoblast function and bone formation (23). Indeed, micro-computed tomography and bone histomorphometry indicated that *Ptx3*^−/−^ mice on B6 background (2.5 months of age) had lower trabecular bone mass than their wild type littermates in long bones and axial skeleton. This phenotype was more obvious in female animals, known to have lower bone formation rate than males of the same age (44, 45). Similar observations were made in the long bones of young *Ptx3*^−/−^ females on SV129 background and aged (6–8 months) *Ptx3*^−/−^ females on B6 background (23).

Further histomorphometric investigations showed no alterations in osteoclast activity, however osteoblast functionality was defective, which resulted into decreased trabecular and endosteal bone formation rate in distal femora of both female and male *Ptx3*^−/−^ mice. In this regard, additional data regarding the cortical compartment and mechanical testing would likely provide further insights into the bone phenotype associated to *Ptx3* deficiency.

The role of PTX3 in bone formation was further evaluated in a tibia mid-diaphyseal fracture model that showed the protein to be made by osteoprogenitor cells, hypertrophic chondrocytes, and active osteoblasts surrounding the fracture gap (23). Specifically, the *Ptx3* gene was found expressed in cells of the non-hematopoietic compartment including α-smooth muscle actin (α-SMA)^+^ osteoprogenitors and CD51^+^ preosteoblasts that populate the soft callus tissue early after bone fracture. These findings were corroborated by immunohistochemistry analyses showing high levels of the PTX3 protein and the osteoblast-specific transcription factors osterix (OSX) and runt-related transcription factor 2 (RUNX2) at the fracture site. Noticeably, proximal to the fracture gap FGF2-positive areas were observed that overlapped with those of osteoprogenitor cells' infiltration, suggesting that PTX3 and FGF2 co-localize and might engage in a complex with potential effects on the FGF2 suppressive activity on osteoblast differentiation (see the “*Ex vivo* and *in vitro*” paragraph in this section). Furthermore, PTX3 was present in the callus during the mineralizing phase, and both percentage of mineralized callus and expression of type 1 collagen were lower in *Ptx3*^−/−^ female mice than in *Ptx3*^+/+^ controls (23).

These observations await confirmatory evidence from other mouse models of physiological bone remodeling and fracture healing, however they are in line with available information from human osteology. In this regard, Scimeca et al. have reported reduced PTX3 expression in the osteoblasts from femoral head biopsies of osteoporotic patients compared to age-matched osteoarthritic patients and young subjects who had undergone post-fracture hip arthroplasty (24). In the same clinical setting, biopsy specimens from osteoporotic patients had reduced trabecular volume (as assessed by histomorphometry) as well as lower expression of RUNX2 and vitamin D receptor (as analyzed by immunohistochemistry). Based on this, the authors proposed PTX3 as a positive regulator of the osteoblast function in physiological conditions, however their conclusions might suffer from the lack of aged-matched control groups without bone pathology in the study design.

### *Ex vivo* and *in vitro*

Human and animal mesenchymal and osteoblast lineage cells express PTX3 at various stages of differentiation (12, 23–27, 35). In a lineage tracing approach, PTX3 expression was documented in murine α-SMA^+^ early osteoprogenitors with proliferative and multi-lineage potential (35). During *in vitro* differentiation of mouse bone marrow-derived stromal cells in osteogenic conditions, the *Ptx3* gene was highly expressed along with osteoblast differentiation markers (i.e., *Osx*, alkaline phosphatase and osteocalcin) (23). In similar osteogenic conditions, the PTX3 protein was found in cultured human bone marrow-derived stromal cells at the preosteoblast stage, and its expression was further increased by TNF-α (27). Moreover, a proteomic study on the human mesenchymal cell secretome indicated up-regulation of PTX3 in human multipotent adipose-tissue derived mesenchymal cells directed to adipogenesis or osteogenesis (26). Finally, human osteoblasts isolated from the trabecular bone of femoral head biopsies expressed high levels of both gene and protein when cultured *in vitro*, and the expression was downregulated in osteoporosis (24).

Despite relatively consistent findings on PTX3 expression in mouse and human osteoblast lineage cells, particularly at the early stages of differentiation, data on the protein's role in osteoblast differentiation are rather conflicting. Treatment of mouse calvarial osteoblasts with different doses of the recombinant protein (0.02–0.47 nM) had no effect on osteoblast proliferation, differentiation, and mineralization (as monitored by alkaline phosphatase, alizarin red, and Von Kossa staining) (27). Similarly, addition of higher concentrations of the exogenous protein (6.25–50 nM) did not change the area covered by colonies expressing alkaline phosphatase in differentiated mouse bone marrow-derived stromal cells. Furthermore, bone marrow-derived osteoprogenitors from *Ptx3*^+/+^ and *Ptx3*^−/−^ mice had similar differentiation potential. Nonetheless, PTX3 (and its N-terminal domain that binds FGF2) reversed the inhibitory effect of FGF2 on osteoblast differentiation, which suggests an indirect effect of the protein on these cells (23).

In contrast to the mouse studies, PTX3 (at 0.47 nM) was shown to accelerate proliferation and hydroxyapatite microcrystal formation in human osteoblasts derived from femoral head biopsies (24). However, these experiments were performed using osteoblasts from osteoporotic patients only at the first or second passage from confluence (~4 weeks of culturing), in the absence of osteogenic stimuli. In addition, in a similar experimental setting, osteoblasts from control subjects (post-fracture hip arthroplasty) underwent significant functional and morphological changes upon treatment with an anti-PTX3 blocking antibody, in particular they acquired a fibroblast-like shape and downregulated the expression of RUNX2 and receptor activator of NF-κB ligand (RANKL) (24).

## Chronic Diseases of the Bone

Several lines of evidence point to PTX3 as a key player in inflammatory diseases, however a few studies only have addressed its contribution to osteoclast activity and inflammation-induced bone loss. In this regard, data are available on periodontitis (28, 46–50), arthritis (30, 51–54), and tumor-associated osteolysis (55–57) (see Table 2). Periodontal infections initiate in the bacterial plaque attached to the tooth surface (mostly Gram-negative anaerobic bacteria), and progress to a chronic disease with irreversible periodontal tissue destruction and osteoclast activation, eventually leading to tooth loss (61, 62). Chronic joint diseases mostly develop as autoimmune processes (i.e., rheumatoid arthritis) or as a result of cartilage damage (i.e., osteoarthritis), and are characterized by joint inflammation and progressive destruction of cartilage and bone (30, 51–54). Malignancies commonly manifest in the skeleton in the form of focal osteolytic lesions associated to metastases, whereby bone resorption (as sustained by osteoclasts) is necessary for tumor cells to grow and invade the mineralized bone (63). Although clinically distinct, these pathological scenarios share a common hallmark, i.e., chronic uncontrolled inflammation with aggravated osteoresorption. In this regard, soluble factors, including acute phase proteins, proinflammatory cytokines and chemokines, antibodies, prostaglandins, tissue-destructive enzymes, and osteoclastogenic mediators collectively participate in the inflammatory process, contributing to bone tissue breakdown. Several pre-clinical and clinical studies point to an association between PTX3 expression and osteoclast activity, which are discussed in the following paragraphs.

### In vivo>

Periodontal disease is often associated to elevation of inflammatory markers both in periodontal tissues and circulation. In a rat model of periodontitis, high levels of the PTX3 protein were found in the gingival tissue and serum, which correlated with alveolar bone resorption and inflammatory cells' infiltration (28). Among other acute phase proteins, PTX3 levels were elevated in the gingival crevicular fluid and plasma of patients with periodontitis, and correlated with the clinical score of the disease (46, 47). Indeed, PTX3 concentration in the gingival tissue of patients with generalized aggressive periodontitis was higher than in those with chronic periodontitis (48, 49). Moreover, recent studies indicated that periodontitis may lead to systemic upregulation of both inflammatory and endothelial dysfunction markers, including PTX3, serum amyloid A (SAA), and amyloid-β peptide (50, 58, 59). In addition to periodontitis, PTX3 (and other inflammatory mediators) was found abundant in the periodontal ligament of the tension zone during orthodontic tooth movement, characterized by enhanced osteoclast activity and rapid bone remodeling (29, 60).

In a mouse model of LPS-induced inflammation, PTX3 expression was found up-regulated in the femoral bone-marrow in areas of increased osteoclast number and osteolytic phenotype (27). Also, PTX3 has been reported to accumulate in the arthritic joints, possibly contributing to the local inflammatory and osteodestructive milieu (30, 51). In a K/BxN serum-transfer arthritis model, PTX3 mRNA and protein levels were both elevated in the ankle joints of arthritic mice, and further increased in arthritic animals lacking matrix metallopeptidase 8, which had more severe disease (30). Expression of PTX3 has also been analyzed in human synovial fluids and tissues from total knee arthroplasty. Immunohistochemistry indicated that the protein co-localizes with endothelial cells and synoviocytes, and is particularly abundant in patients with rheumatoid arthritis, as compared to those with osteoarthritis and post-traumatic effusion (51). Increased levels of this pentraxin were found in the synovial fluid of patients with different clinical forms of juvenile idiopathic arthritis, with a positive correlation with disease severity and progression (54).

In the context of tumor-associated osteolysis, PTX3 expression (based on public genome-wide gene expression data) has been reported to be up-regulated in the distant bone metastases of breast cancer as compared to lung, liver and brain metastases (56, 57), and this has been linked to enhanced osteolysis (see below).

### *Ex vivo* and *in vitro*

*In vitro* expression of *Ptx3* has been documented in osteoclastogenic cultures of bone marrow cells stimulated with RANKL and monocyte/macrophage colony-stimulating factor (M-CSF) (23). In this setting, addition of the exogenous protein (0.02–50 nM) did not change the number of differentiated osteoclasts expressing tartrate-resistant acid phosphatase (23, 27). However, Lee et al. have reported that PTX3 had an indirect osteoclastogenic effect by increasing the RANKL/osteoprotegerin (OPG) ratio in mouse calvarial preosteoblasts but not in mature osteoblasts (27). In co-cultures of mouse preosteoblasts and bone marrow cells (stimulated with vitamin D_3_ and prostaglandin E_2_), PTX3 (0.02–0.47 nM) enhanced osteoclast differentiation, and *Ptx3* silencing (by siRNA) in preosteoblasts had opposite effects. Addition of TNF-α further increased the number of differentiated osteoclasts, a process that was counteracted by *Ptx3* gene silencing (27). However, an active role of other bone marrow cells (that are present in the applied co-culture system) cannot be ruled out.

Inflammatory cytokines, particularly TNF-α, have been described to induce PTX3 expression in cultured human bone marrow-derived preosteoblasts (27), osteoarthritic synoviocytes (51), and synovial cell lines (54). Moreover, synoviocytes from patients with rheumatoid arthritis constitutively express high levels of PTX3 mRNA and protein *in vitro* (51, 52), and these were not affected by neutralization of TNF-α or IL-1β (51).

Breast and gastric cancer cell lines have been reported to express PTX3, and the exogenous protein promoted migration of breast cancer cells and macrophages (56, 57). In an *in vitro* system where a human breast cancer cell line and a mixture of mouse calvarial osteoblasts and bone marrow derived macrophages were co-cultured in the upper and lower chambers of a transwell, respectively, stimulation of PTX3 expression in the tumor cells by TNF-α enhanced *Rankl* expression and osteoclast formation in the lower compartment, suggesting a role for PTX3 in cancer-related osteolysis that however requires validation *in vivo* (57).

## Concluding Remarks

**Figure 1:**
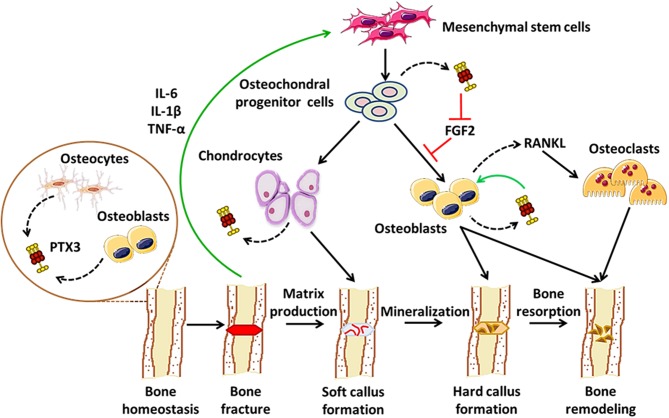
Proposed roles of PTX3 in bone homeostasis and fracture healing. In conditions of physiological bone turn-over, PTX3 is expressed by osteoblasts and bone-encased osteocytes, likely contributing to bone deposition via yet unknown mechanisms (24). Following fracture, inflammatory mediators (e.g., IL-6, IL-1β, TNF-α), along with other factors, promote osteoblast development, and differentiation. In these conditions, PTX3 (made by osteochondral progenitor cells, chondrocytes, and osteoblasts) reverses the inhibitory effects exerted by FGF2 on osteoblast differentiation, thereby contributing to matrix mineralization (23). In the late stages of fracture healing, PTX3 likely participates in bone remodeling by stimulating RANKL production and osteoclastogenesis (27).

As a paradigmatic component of the humoral arm of innate immunity, PTX3 exerts a number of functions at the crossroad between host-pathogen interface, inflammation, and matrix remodeling (6). Recent evidence points to contrasting roles for PTX3 in bone pathophysiology: on one hand, it acts as a promoter of osteoblast differentiation and mineral matrix deposition (23); on the other, it supports osteoclastogenesis in inflammatory conditions, including arthritis and bone metastasis (27, 56, 57). Furthermore, PTX3 has been associated with periodontal tissue inflammation, a condition that precedes alveolar bone resorption (52–57). These pleiotropic effects possibly derive from inherent differences in the applied experimental models, and are likely amplified by the structural complexity of the PTX3 protein that supports a multiplicity of interactions, thereby context-dependent functions. In this regard, we propose a model that accounts for stage-specific expression and activity of this pentraxin in physiological bone remodeling and fracture healing, i.e., osteoblast-derived PTX3 likely contributes to bone growth, the inflammatory response that follows bone fracture leads to up-regulation of the gene in osteochondral progenitor cells, chondrocytes and osteoblasts, where the newly made protein has FGF2-dependent matrix mineralization promoting effects, in the late phases of fracture healing PTX3 participates in bone remodeling via stimulation of RANKL synthesis and osteoclastogenesis (see Figure 1). This information notwithstanding, little is known regarding bone-related effects of the interaction between PTX3 and its cognate ligands (besides FGF2) with established properties in bone physiopathology, for examples the complement system (64). Also, given the prominent protective role of PTX3 in the resistance to selected microbial pathogens, assessing its function in bone infections other than periodontitis (e.g., osteomyelitis) is relevant and deserves further investigations, owing to its potential application in human diseases.

## Author Contributions

AI and DG wrote the manuscript. RP took care of the tables, figure, and legend. CS, BB, and AM contributed to critical revision.

### Conflict of Interest

The authors declare that the research was conducted in the absence of any commercial or financial relationships that could be construed as a potential conflict of interest.
